# Towards a universal mechanism for successful deep learning

**DOI:** 10.1038/s41598-024-56609-x

**Published:** 2024-03-11

**Authors:** Yuval Meir, Yarden Tzach, Shiri Hodassman, Ofek Tevet, Ido Kanter

**Affiliations:** 1 Department of Physics, Bar-Ilan University, 52900 Ramat-Gan, Israel; 2 Gonda Interdisciplinary Brain Research Center, Bar-Ilan University, 52900 Ramat-Gan, Israel

**Keywords:** Information theory and computation, Statistical physics, thermodynamics and nonlinear dynamics, Computer science

## Abstract

Recently, the underlying mechanism for successful deep learning (DL) was presented based on a quantitative method that measures the quality of a single filter in each layer of a DL model, particularly VGG-16 trained on CIFAR-10. This method exemplifies that each filter identifies small clusters of possible output labels, with additional noise selected as labels outside the clusters. This feature is progressively sharpened with each layer, resulting in an enhanced signal-to-noise ratio (SNR), which leads to an increase in the accuracy of the DL network. In this study, this mechanism is verified for VGG-16 and EfficientNet-B0 trained on the CIFAR-100 and ImageNet datasets, and the main results are as follows. First, the accuracy and SNR progressively increase with the layers. Second, for a given deep architecture, the maximal error rate increases approximately linearly with the number of output labels. Third, similar trends were obtained for dataset labels in the range [3, 1000], thus supporting the universality of this mechanism. Understanding the performance of a single filter and its dominating features paves the way to highly dilute the deep architecture without affecting its overall accuracy, and this can be achieved by applying the filter’s cluster connections (AFCC).

## Introduction

A prototypical supervised learning task involves object classification, which is realized using deep architectures^1–3^. These architectures consist of up to hundreds of convolutional layers (CLs)^4–6^, each of which consists of tens or hundreds of filters, and several additional fully connected (FC) hidden layers. As the classification task becomes more complex, a small training dataset and distant objects that belong to the same class, deeper architectures are typically required to achieve enhanced accuracies. The training of their enormous number of weights requires nonlocal training techniques such as backpropagation (BP)^7–9^, which are implemented by advanced GPUs, and can guarantee convergence to a suboptimal solution only.

The current knowledge of the underlying mechanism of successful deep learning (DL) is vague^1,10–13^. The common assumption is that the first CL reveals a local feature of an input object, where large-scale features and features of features, which characterize a class of inputs, are progressively revealed in the subsequent CLs^1,14–17^. The terminologies of the features and features of features and the possible hierarchy among them have not been quantitatively well defined. In addition, the existence of the underlying mechanism of successful DL remains unclear. Is the realization of a classification task using deep and shallower architectures with different accuracies based on the same set of features? Similarly, is the realization of different classification tasks using a given deep architecture based on the same type of features?

A quantitative method to explain the underlying mechanism of successful DL^18^ was recently presented and exemplified using a limited deep architecture and dataset, namely VGG-16^10^ on CIFAR-10^14^ and advanced variants thereof^10,19^. This method enables the quantification of the progressive accuracies with the layers and the functionality of each filter in a layer, and consists of the following three main stages.

In the first stage, the entire deep architecture is trained using optimized parameters to minimize the loss function. In the second stage, the weights of the first $m$ trained layers remain unchanged and their outputs are FC with random initial weights to the output layer, which represent the labels. The output of the first $m$ layers represents the preprocessing of an input using the partial deep architecture and the FC layer is trained to minimize the loss, which is a relatively simple computational task. The results indicate that the test accuracy^20^ increases progressively with the number of layers towards the output.

**Figure 1:**
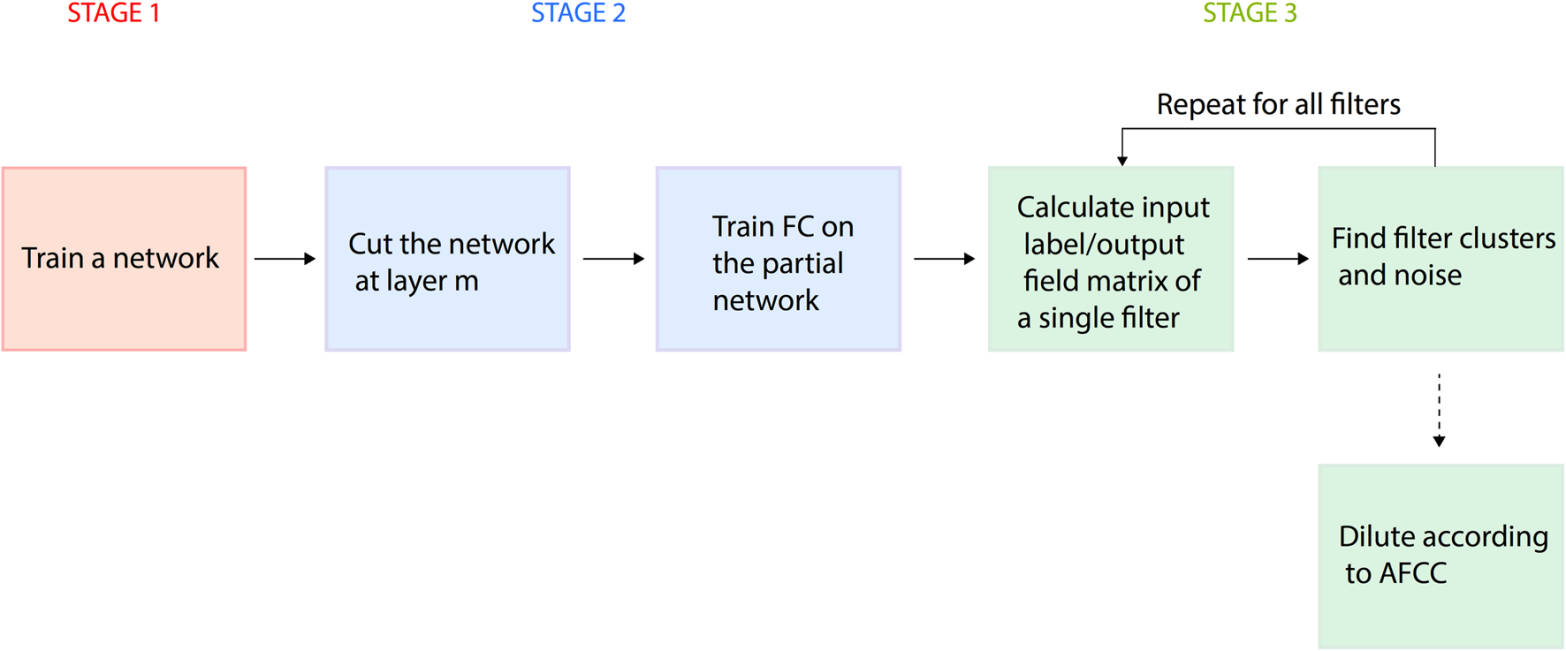
Flowchart of the three stages for calculating the performance of a single filter. The entire deep network is trained to minimize the loss function (Stage 1). The $m$th layer is FC to the output and is then trained to minimize the loss with fixed weights of the previous $m$ layers (Stage 2). The properties of a specific filter are calculated by silencing all the weights except those emerging from that specific filter. The matrix elements representing the average output field on an output unit for a specific input label are calculated using the training dataset. The clusters and noise elements of each filter are then calculated using the matrix elements. Finally, learning using a diluted deep architecture in accordance with the calculated clusters, namely the AFCC method, is performed.

In the third stage, the trained weights of the FC layer are used to quantify the functionality of each filter constituting its input layer. The single-filter performance is calculated with all weights of the FC layer silenced except for the specific weights that emerge from a single filter. At this point, the test inputs are presented and preprocessed by the first $m$ layers, but influence the output units only through the small aperture of one filter. The results demonstrate that each filter essentially identifies a small subset among the ten possible output labels, which is a feature that is progressively sharpened with the layers, thereby resulting in enhanced signal-to-noise ratios (SNRs) and accuracies^18^. These three stages, which constitute the method by which the performance of a single filter is calculated, are presented in Fig. 1.

As the method for the underlying mechanism of successful DL was tested for only one deep architecture and one dataset composed of small images^18^, its generality is questionable. In this study, we investigate its universality by training EfficientNet-B0^21^ and VGG-16 on extended datasets where the number of output labels is in the range of $\left[3, 1000\right],$ taken from CIFAR-10^14^, CIFAR-100^14^ and ImageNet^15,22^. The results strongly suggest the universality of the proposed DL mechanism, which is verified for varying numbers of output labels with three orders of magnitudes, small ($32\times 32$) and large ($224\times 224$) images, and state-of-the-art deep architectures.

In the following section, the underlying mechanism of DL is explained using the results for VGG-16 on CIFAR-100. Thereafter, the results are extended to EfficientNet-B0 on CIFAR-100 and ImageNet. Finally, the case of training VGG-16 and EfficientNet-B0 on varying number of labels taken from CIFAR-100 as well as VGG-16 on CIFAR-10 is discussed. Subsequently, a summary and several suggested techniques for improving the computational complexity and accuracy of deep architectures are briefly presented in the “Discussion” section.

## Results

### Results of VGG-16 on CIFAR-100

**Figure 2:**
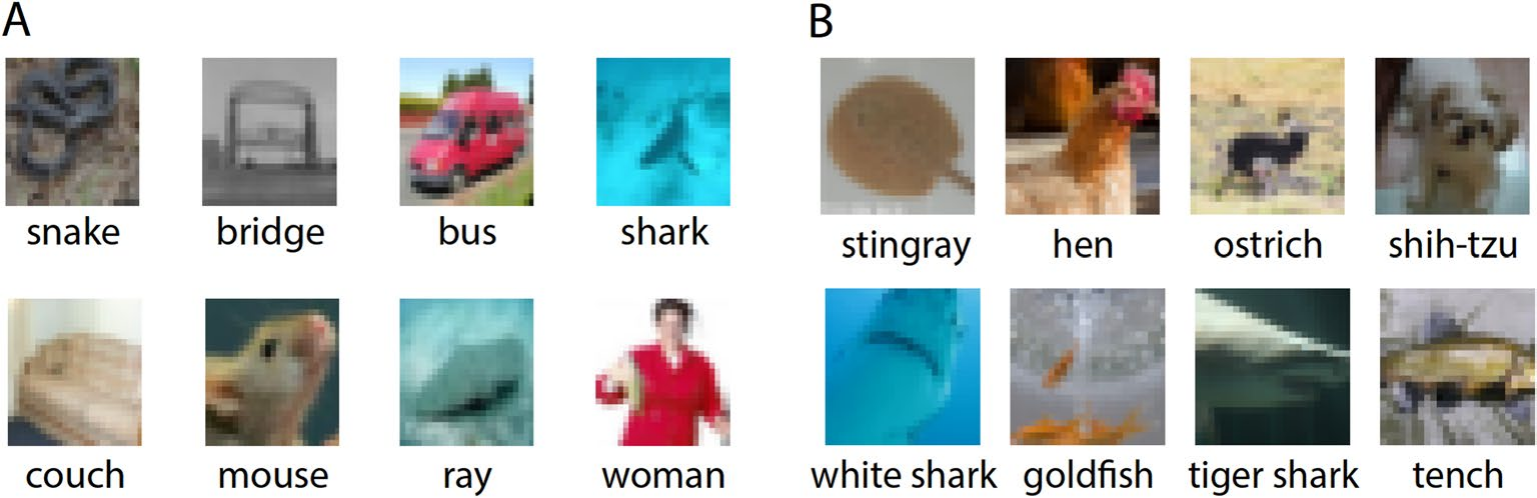
Image samples of the datasets. (**A**) Eight image samples with different labels from the CIFAR-100 dataset. (**B**) Eight image samples with different labels from the ImageNet dataset.

The training of VGG-16 on CIFAR-100 (Fig. 2A) with optimized parameters yielded a test accuracy of approximately 0.75 (Table 1 and Supplementary Information), which was slightly higher than the previously obtained accuracy^23^. Next, the weights of the first $m$ trained layers were held unchanged, and their outputs were FC with random initial weights to the output layer. The selected layers were those that terminated with max-pooling, $m = 2, 4, 7, 10,$ and $13$. The training of these FC layers indicates that the accuracy increased progressively with the number of layers and saturated at $m=10$, (Table 1), which is a result of the small image inputs of $32\times 32$. The three CLs $(3\times 3)$, layers $8{-}10,$ generate a $7\times 7$ receptive field^24^ covering a filter size of $4\times 4$. Hence, layers $11{-}13$ are redundant for small images.

**Table 1:** Accuracy per layer and statistical features of their filters for VGG-16 trained on CIFAR-100.

VGG-16 on CIFAR-100							
Layer	${N}_{f}$	${F}_{s}$	$F{C}_{s}$	Accuracy	$n$	${N}_{c}$	${C}_{s}$
13	512	1 × 1	512	0.745	277.1	1.7	7.7
10	512	2 × 2	2048	0.752	16.3	2.6	2.0
7	256	4 × 4	4096	0.577	117.9	2.8	2.7
4	128	8 × 8	8192	0.439	552.4	5.1	2.8
2	64	16 × 16	16,384	0.352	987.8	5.8	3.2

${N}_{f}$ number of filters of layers terminating with max-pooling, ${F}_{s}$ filter sizes, $F{C}_{S}$ size of trained FC layer connected to the output units, $n$ average noise per filter, ${N}_{c}$ average number of clusters per filter, ${C}_{S}$ average cluster size.

**Figure 3:**
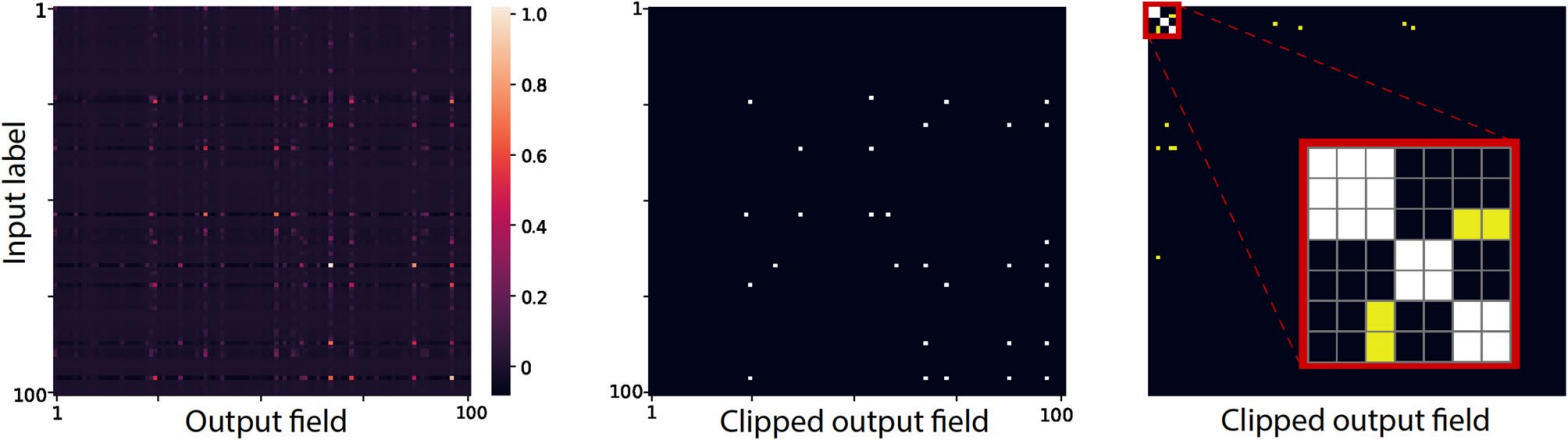
Single filter performance. Left: the matrix element (*i*,*j*) of a filter belonging to layer 10 of VGG-16 trained on CIFAR-100 represents the averaged fields that were generated by label *i* test inputs on an output *j*, where the matrix elements were normalized by their maximal element. Middle: the Boolean clipped matrix (0/1 is represented by black/white pixels) following a given threshold. Right: permutations of the clipped matrix labels resulting in three diagonal clusters: two $2\times 2$ and one $3\times 3$ (magnified upper-left corner red box), where above-threshold $n$ elements out of the cluster are noise elements, denoted by yellow.

The performance of a single filter is represented by a $100\times 100$ matrix and is exemplified for layer $10$ (Fig. 3, left). The element $(i, j)$ represents the average of the fields that are generated by the label $i$ test inputs on output $j$, where the matrix elements are normalized by their maximal element. Next, its Boolean clipped matrix following a specified threshold is calculated (Fig. 3, middle) as well as its permuted version to form diagonal clusters (Fig. 3, right, Supplementary Information). The above-threshold elements out of the diagonal clusters are defined as the filter noise $n$ (yellow elements in Fig. 3, right).

The performance of each filter was calculated using test inputs, with all weights of the trained FC layer silenced except for those that emerged from the filter. The estimated main averaged properties of the ${N}_{f}(m)$ filters belonging to the *m*th layer are the cluster size ${C}_{s}(m)$, number of clusters per filter ${N}_{c}\left(m\right),$ and number of noise elements out of the clusters $n(m)$ (Table 1). The results clearly indicate that $n(m)$ decreases with $m$ until the accuracy is saturated at $m=10$, where the average cluster size is small at $2$ out of $100$ labels. In addition, the average number of cluster elements is very small, ${{N}_{c}\cdot C}_{s}^{2}=$ $2.6\times {2}^{2}=10.4$ out of the ${10{,}000}$ matrix elements (Table 1).

**Figure 4:**
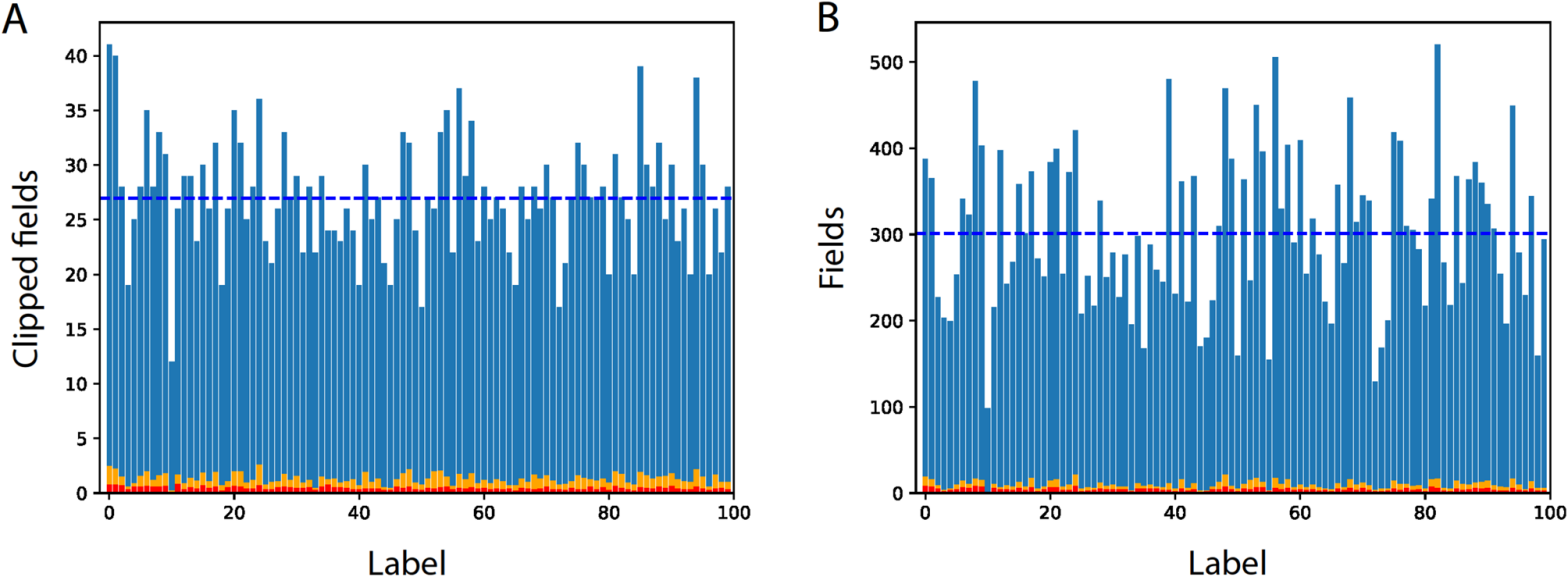
Comparison of SNRs obtained from above-threshold Boolean filters and their fields. (**A**) The signal per label (blue), $nois{e}_{I}$ per label (red), and $nois{e}_{I}+nois{e}_{E}$ per label (orange) (Eqs. (1–4)) that were obtained from the above-threshold clipped Boolean fields of the $512$ filters of the 10th layer of VGG-16 trained on CIFAR-100. The average signal (dashed blue horizontal line), $nois{e}_{I}$ (red), and $nois{e}_{I}+nois{e}_{E}$ (orange) are $26.95, 0.46$ and $1.3$, respectively, which are similar to the estimated values obtained from Eqs. (1–3). (**B**) Similar to (**A**), using the fields of the above-threshold elements of the filters. The average signal (dashed blue horizontal line), $nois{e}_{I}$ (red), and $nois{e}_{I}+nois{e}_{E}$ (orange) values are $301, 4.7$, and $9.7$, respectively.

The estimation of the SNR using the following quantities is required to understand the mechanism underlying DL. The average appearance number of each label among the ${N}_{l}$ labels in the clusters of the layer is

1 $$signal={(C}_{s}\cdot {N}_{c}\cdot {N}_{f})/{N}_{l },$$

which represents the $signal$ under the assumption of uniform number of appearances of each diagonal element over all clusters. The average expected $signal$ that emerges from the 10th layer is approximately $26.6$ (Table 1 and Eq. (1)), which fluctuates among the $100$ labels (Fig. 4A). The average internal cluster noise, $nois{e}_{I}$, is equal to the average number of appearances of other labels in the clusters forming the $signal$ of a given label,

2 $$nois{e}_{I}=\frac{\left({C}_{s}-1\right)}{{N}_{l}-1}\cdot signal ,$$

which results in an average $nois{e}_{I}$ of approximately $0.27$ for the 10th layer, with relatively small fluctuations among the labels (Fig. 4A). Furthermore, $SN{R}_{I}=$ $\frac{signal}{nois{e}_{I}}\gg 1$ provided that $\frac{{C}_{s}}{{N}_{l}}\ll 1$.

The second type of noise stems from the above-threshold matrix elements out of the clusters, which is the external noise $n.$ Using the assumption of uniform noise over the off-diagonal matrix elements, the average value of this noise is approximated as follows:

3 $$nois{e}_{E}=n\cdot \frac{{N}_{f}}{{\left({N}_{l}\right)}^{2}} ,$$

where the average number of elements that belong to the clusters of each filter is negligible compared to ${\left({N}_{l}\right)}^{2}$ (Fig. 3). As $nois{e}_{E}\propto n$,

4 $$SN{R}_{E}=\frac{signal}{nois{e}_{E}}={(C}_{s}\cdot {N}_{c}\cdot {N}_{l})/n,$$

which increases with a decrease in $n$. This is the origin of the DL mechanism, where $n$ decreases progressively with the number of layers, thereby enhancing the accuracy (Eq. (4)). For example, $nois{e}_{E}$ is approximately $0.83$ for the 10th layer, whereas it is approximately $29$ for the 4th layer where the signal is only $18$ (Table 1 and Eqs. (1)–(3)). Note that the above calculations neglect the subthreshold elements; however, they are typically several orders of magnitude smaller than the above-threshold elements and are frequently negative^18^ (Fig. 3).

Although the above estimations of $SN{R}_{I}$ and $SN{R}_{E}$, Eqs. (1–4), were expected to fluctuate among the labels, they were found to be much greater than unity per label (Fig. 4A). In addition, these SNRs may be far from reality because the matrix (Fig. 3, left) was first normalized by its maximal value, which varied significantly among the filters, following which the above-threshold elements were defined to form a Boolean matrix. Nevertheless, the summation of the fields of the above-threshold elements, instead of their Boolean summations, indicates that $SN{R}_{I}$ and $SN{R}_{E}$ for each label were much greater than unity (Fig. 4B), and their averaged values are comparable to the estimated values based on the Boolean filters.

The progressive decrease in $nois{e}_{E}$ with the layers of a given trained deep architecture is the underlying mechanism for successful DL (Eq. (4)). Nevertheless, a large estimated $SN{R}_{E}$ does not necessarily ensure an accuracy that approaches unity because it is based only on averaged quantities ((Eqs. (1–4)), where large fluctuations around their average values are expected, particularly for large ${N}_{l}$. In addition, a positive field of a cluster element cannot exclude negative fields for a large fraction of the corresponding input label.

### Results of EfficientNet-B0 on CIFAR-100

The training of the expanded $224\times 224$ images^25^ of CIFAR-100 on EfficientNet-B0 was performed using transfer learning^26,27^ (Supplementary Information) and yielded an improved accuracy of $0.867$ (Table 2). This architecture does not include max-pooling operators, and a decrease of a factor of two in the layer dimensions is achieved using stride-2 at specific CLs. Hence, similar to the case of VGG-16, the accuracies and average filter properties were estimated at the end of the stages with stride-2, $1, 3, 4, 5, 7,$ and $9$. The outputs of these stages were first sampled by $7\times 7$ average pooling as built-in in stage $9$, followed by a layer that was FC to the $100$ output units which was trained to minimize the loss (Supplementary Information). The results indicate that the accuracy almost always increased with the number of stages and the noise per filter decreased (Table 2), thereby supporting the proposed universal mechanism underlying DL. The semi-plateau of the accuracies of stages $4$ and $5$ was common to all examined datasets using EfficientNet-B0, which suggests that this architecture might be simplified without affecting its accuracy by removing, for example, some layers around stage $5$ (see “Discussion” section).

**Table 2:** Accuracy per stage and statistical features of their filters for EfficientNet-B0 trained on CIFAR-100.

EfficientNet-B0 on CIFAR-100							
Stage	${N}_{f}$	${F}_{s}$	$F{C}_{s}$	Accuracy	$n$	${N}_{c}$	${C}_{s}$
9	1280	1 × 1	1280	0.867	31.6	1.2	3
7	192	1 × 1	192	0.729	232.0	3.5	2.1
5	80	2 × 2	320	0.503	308.7	3.6	1.7
4	40	4 × 4	640	0.502	436.9	5.2	1.7
3	24	8 × 8	1536	0.426	526.2	5.6	1.8
1	32	16 × 16	8192	0.259	1208.1	2.9	5.3

The presented results were obtained at the end of stages consisting of stride-2 only, reducing by factor two the size of the output layer, similar to the max-pooling operator in VGG-16 (Table 1).

The progressive decrease in the noise $n$ with the layers or stages of a particular deep architecture is the underlying mechanism of DL. However, a comparison of the SNRs of two deep architectures does not necessarily correlate with accuracies. For instance, the improved EfficientNet-B0 accuracy of $0.867$, in comparison with $\sim 0.75$ for VGG-16 (Tables 1, 2), could not be simply deduced from their SNRs (Eq. (4)) because $nois{e}_{E}$ was doubled for EfficientNet-B0, whereas ${C}_{s}\cdot {N}_{c}$ was reduced from $5.2$ in VGG-16 to only approximately $4$. The accuracy improvement of EfficientNet-B0 probably stems from the enhanced $signal$ of approximately $64$, whereas it was only approximately $27$ for VGG-16 (Eq. (1)), as well as the distribution of their output fields for the test inputs.

### Results of EfficientNet-B0 on ImageNet

The presented underlying mechanism of DL was extended to a dataset consisting of $1000$ labels and $224\times 224$ input images, with the pre-trained EfficientNet-B0 on the ImageNet dataset^15,22^ (Fig. 2B) constituting the initial stage of the following procedure. The output layer of stages $1, 3, 4, 5, 7$, and $9$ was FC with random initial weights to the $1000$ outputs (Table 3). Next, these FC weights were trained to minimize the loss, with all remaining weights of the trained EfficientNet-B0 kept fixed. Finally, the accuracy of the different stages and statistical properties of their filters were estimated (Table 3).

**Table 3:** Accuracy per stage and statistical features of their filters for EfficientNet-B0 trained on ImageNet.

EfficientNet-B0 on ImageNet							
Stage	${N}_{f}$	Filter’s outputs	FC size	Accuracy	$n$	${N}_{c}$	${C}_{s}$
9	1280	1 × 1	1280	0.750	1057.7	3.5	4.4
7	192	1 × 1	192	0.489	5729.6	8.3	3.2
5	80	2 × 2	320	0.187	7168.0	8.9	2.6
4	40	4 × 4	640	0.136	13,416.9	16.5	2.5
3	24	8 × 8	1536	0.065	6471.0	13.2	1.9
1	32	16 × 16	8192	0.022	50,381.9	8.5	7.4

The presented results were obtained at the end of stages consisting of stride-2 only, similar to Table 2.

As training of these FC layers using the large ImageNet dataset ($1.4M$ images) was beyond our computational capability, we divided the ${50{,}000}$ images from the validation test into ${40{,}000}$ images for training and ${10{,}000}$ for testing. This training of the stage 9 FC layer was similar to transfer learning^26,27^ and yielded an accuracy of approximately $0.75$, where the original accuracy of the entire pre-trained EfficientNet-B0 was approximately $0.78$ (Supplementary Information).

The accuracy increases with the stages, whereas the noise $n$ typically decreases (Table 3), which supports the universal underlying mechanism of DL. Interestingly, the average cluster size, ${C}_{s},$ and number of clusters per filter, ${N}_{c}$, which were measured at the last stage or layer that saturated the accuracy, increased only slightly while ${N}_{l}$ increased from $100$ to $1000$ (Tables 1, 2, 3). The exception of stage $3$ in which $n$ was non-monotonic (Table 3) may stem from the small ${N}_{f} = 24$, resulting in ${N}_{f}\cdot {N}_{c}\cdot {C}_{s}\sim 601<1000$, whereas it was greater than $1000$ for other stages. For stage $3$, a large fraction of the labels ($\sim 500$) did not appear in any of the clusters and their estimated signal was zero. For all other stages, ${N}_{f}$ was larger and ${N}_{f}\cdot {N}_{c}\cdot {C}_{s}>1000$, resulting in significantly lower number of labels with zero signal. Note that this anomaly of stage $3$ was indeed absent in CIFAR-100 (Table 1).

Similar trends are expected for VGG-16 on ImageNet with much lower accuracy and higher noise than EfficientNet-B0. In this case, the image dimension is greater by a factor of 7; hence, the FC layer sizes become significantly larger, and the optimization of those layers is currently beyond our computational capabilities.

### Datasets with varying number of labels

#### CIFAR-100 with varying number of labels

**Figure 5:**
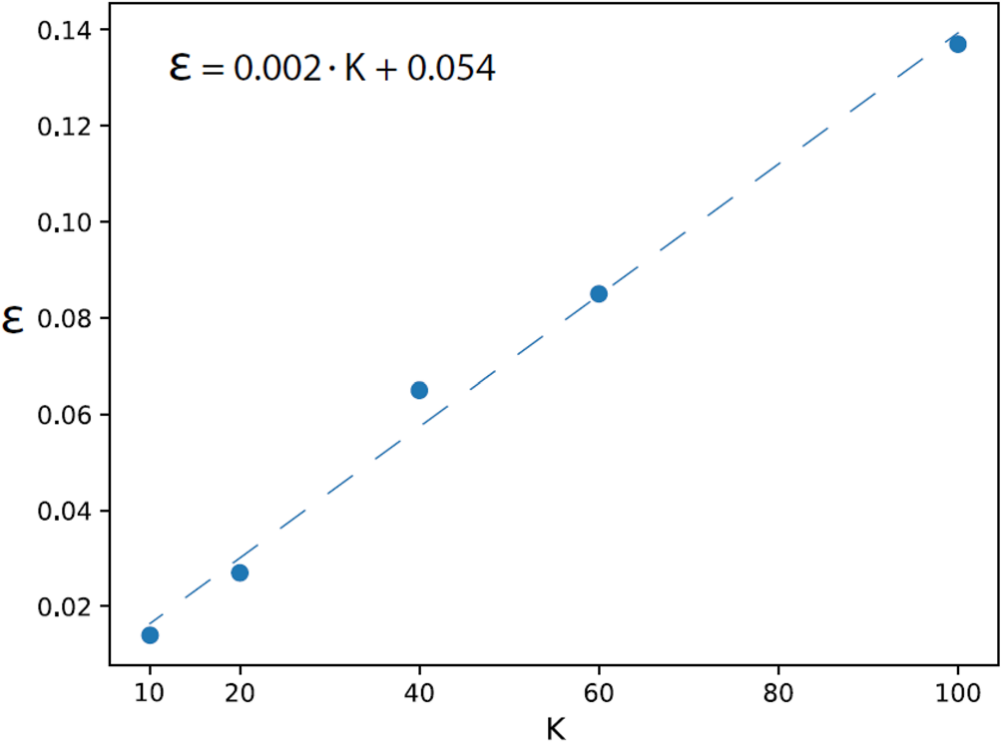
Test error for VGG-16 trained on CIFAR-K/100. Test error$, \epsilon =1-accuracy$, obtained at 10th layer of VGG-16 trained on $K$ labels from CIFAR-100, namely CIFAR-K/100, and the linear fit approximation (dashed line). The subset of $K$ labels included smaller ($<K$) selected labels (Supplementary Information).

The proposed universal mechanism for DL was extended by varying the output labels $K$ out of $100$ in CIFAR-100, where $K=10, 20, 40,$ and $60$. The results for VGG-16 are summarized in Table 4, and indicate similar trends to those observed for $K=100$ (Table 1). The accuracy increased progressively with the number of layers until saturation at the 10th layer, and the out-of-cluster noise $n$ decreased progressively with the number of layers. Interestingly, ${C}_{s}$ and ${N}_{c}$ were only slightly affected by $K$ at the 10th layer (Tables 1, 4). The test error, $\epsilon =1-accuracy$, is expected to increase with $K$ since the classification task is more complex; the results indicate that this increase is approximately linear with $K$ (Fig. 5). Nevertheless, the extrapolation of the linear fit to a smaller $K$ approaching unity indicates that a limited crossover is expected, as $\epsilon$ is expected to vanish for $K=1$.

**Table 4:** Accuracy per layer and statistical features of their filters for VGG-16 trained on $K$ labels from CIFAR-100.

Layer	${N}_{f}$	${F}_{s}$	$F{C}_{s}$	Accuracy	$n$	${N}_{c}$	${C}_{s}$
VGG-16 on CIFAR-10/100							
13	512	1 × 1	512	0.926	3.26	1.01	2.2
10	512	2 × 2	2048	0.931	4.86	1.83	1.6
7	256	4 × 4	4096	0.908	10.11	1.47	1.7
4	128	8 × 8	8192	0.890	15.83	1.6	1.8
2	64	16 × 16	16,384	0.829	18.64	1.6	2.0
VGG-16 on CIFAR-20/100							
13	512	1 × 1	512	0.9115	9.92	1.02	3.7
10	512	2 × 2	2048	0.9115	13.6	2.33	1.9
7	256	4 × 4	4096	0.9065	33.6	1.64	2.31
4	128	8 × 8	8192	0.8465	57	2	2.4
2	64	16 × 16	16,384	0.752	68.23	1.83	2.7
VGG-16 on CIFAR-40/100							
13	512	1 × 1	512	0.8553	51.8	1.11	7.5
10	512	2 × 2	2048	0.8567	12.3	2.92	2
7	256	4 × 4	4096	0.7825	38.4	2.44	2.17
4	128	8 × 8	8192	0.6388	143.8	3.22	2.54
2	64	16 × 16	16,384	0.5380	203.6	3.5	2.7
VGG-16 on CIFAR-60/100							
13	512	1 × 1	512	0.8277	123.9	1.3	8.13
10	512	2 × 2	2048	0.8275	18.17	2.78	2.3
7	256	4 × 4	4096	0.7148	39.52	2.16	2.24
4	128	8 × 8	8192	0.5392	260.6	4.16	2.6
2	64	16 × 16	16,384	0.4480	423.92	4.5	3

The results are similar to those of Table 1, where VGG-16 was trained on $K=10, 20, 30,$ and $60$ labels out of $100,$ namely CIFAR-K/100 (Supplementary Information).

**Figure 6:**
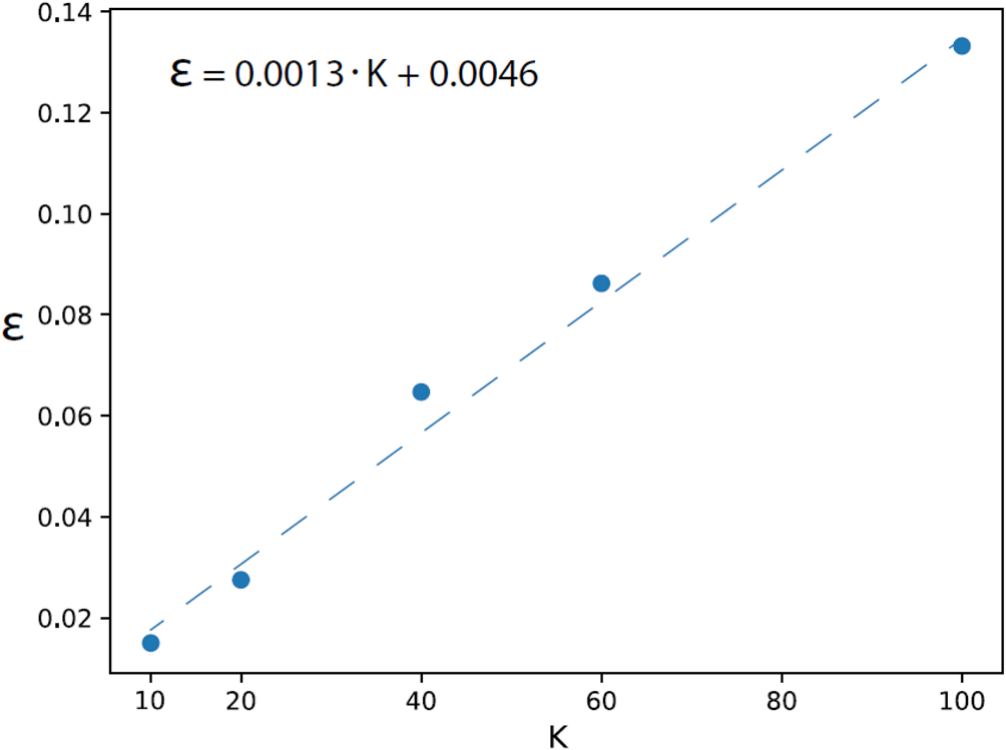
Test error for EfficientNet-B0 trained on CIFAR-K/100. Average test error$,\epsilon =1-accuracy$, obtained at stage $9$ of EfficientNet-B0 trained on $K$ labels from CIFAR-100 (similar to Fig. 5) and the linear fit approximation (dashed line).

Similar trends were observed for EfficientNet-B0 trained with $K=10, 20, 40,$ and $60$ labels from CIFAR-100 (Table 5). Again, the accuracy increased progressively with the stages (except for stage $5$ at $K=60$) and $n$ decreased progressively with the stages, thereby exemplifying the universality of the mechanism underlying DL. Similar to the case of VGG-16, the test error $\epsilon$ also increased approximately linearly with $K$ and almost vanished, as expected, at $K=1$ (Fig. 6). Note that the slope of the approximated linear fit fluctuated slightly among the samples (Supplementary Information). In addition, the average cluster size ${C}_{s}$ increased slightly from $1.6$ for $K=10$ to $3$ for $K=100$, whereas the number of clusters per filter ${N}_{c}$ was approximately $1.1$ and independent of $K$ (Table 5).

**Table 5:** Accuracy per layer and statistical features of their filters for EfficientNet-B0 trained on $K$ labels from CIFAR-100.

Stage	${N}_{f}$	${F}_{s}$	$F{C}_{s}$	Accuracy	$n$	${N}_{c}$	${C}_{s}$
EfficientNet-B0 on CIFAR-10/100							
9	1280	1 × 1	1280	0.986	3.8	1.08	1.6
7	192	1 × 1	192	0.955	8.3	1.80	1.3
5	80	2 × 2	320	0.851	10.6	1.85	1.2
4	40	4 × 4	640	0.845	12.8	2.15	1.3
3	24	8 × 8	1536	0.755	14.5	2.75	1.3
1	32	16 × 16	8192	0.634	18.1	1.55	1.9
EfficientNet-B0 on CIFAR-20/100							
9	1280	1 × 1	1280	0.973	8.1	1.1	2.0
7	192	1 × 1	192	0.915	22.9	2.1	1.5
5	80	2 × 2	320	0.765	29.7	2.0	1.3
4	40	4 × 4	640	0.764	40.8	2.8	1.4
3	24	8 × 8	1536	0.645	48.1	3.4	1.4
1	32	16 × 16	8192	0.482	63.8	1.5	3.1
EfficientNet-B0 on CIFAR-40/100							
9	1280	1 × 1	1280	0.935	16.4	1.1	2.4
7	192	1 × 1	192	0.849	64.2	2.6	1.7
5	80	2 × 2	320	0.652	85.2	2.7	1.4
4	40	4 × 4	640	0.650	111.3	3.3	1.5
3	24	8 × 8	1536	0.553	129.6	3.9	1.6
1	32	16 × 16	8192	0.362	223.6	1.9	3.9
EfficientNet-B0 on CIFAR-60/100							
9	1280	1 × 1	1280	0.915	21.4	1.2	2.6
7	192	1 × 1	192	0.810	121.8	3.2	1.9
5	80	2 × 2	320	0.593	152.0	3.0	1.6
4	40	4 × 4	640	0.603	200.0	3.8	1.6
3	24	8 × 8	1536	0.511	252.3	4.8	1.7
1	32	16 × 16	8192	0.313	492.6	2.5	4.4

The results here are similar to those of Table 2, where EfficientNet-B0 was trained on $K=10, 20, 30,$ and $60$ labels out of 100, namely CIFAR-K/100 (Supplementary Information).

#### CIFAR-10 with varying number of labels

**Figure 7:**
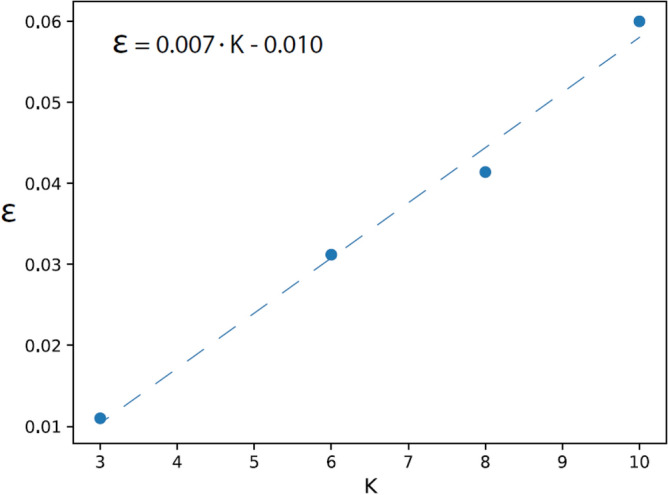
Test error for VGG-16 trained on CIFAR-K/10. Test error$,\epsilon =1-accuracy$, obtained at the 10th layer of VGG-16 trained on $K$ labels from CIFAR-10 and the linear fit approximation (dashed line).

The universal mechanism of DL was also verified for VGG-16 trained on CIFAR-10 with varying $K=3, 6, 8,$ and $10$ (Table 6). The accuracy increased progressively with the number of layers until saturated at the 10th layer, and $n$ decreased progressively with the number of layers. Similar to the case of CIFAR-100, the test error increased approximately linearly with $K$ (Fig. 7), where the extrapolation for $K=1, \epsilon$ approaches zero, as expected.

**Table 6:** Accuracy per label and statistical features of their filters for VGG-16 trained on $K$ labels from CIFAR-10.

Layer	${N}_{f}$	${F}_{s}$	$F{C}_{s}$	Accuracy	$n$	${N}_{c}$	${C}_{s}$
VGG-16 on CIFAR-3/10							
13	512	1 × 1	512	0.988	0.07	1	1.02
10	512	2 × 2	2048	0.988	0.27	1.5	1.02
7	256	4 × 4	4096	0.989	0.67	1.2	1.06
4	128	8 × 8	8192	0.972	1.70	1.1	1.12
2	64	16 × 16	16,384	0.927	1.78	1.1	1.25
VGG-16 on CIFAR-6/10							
13	512	1 × 1	512	0.968	0.40	1	1.8
10	512	2 × 2	2048	0.967	1.16	2.4	1.3
7	256	4 × 4	4096	0.957	2.12	1.3	1.4
4	128	8 × 8	8192	0.930	6.69	1.2	1.6
2	64	16 × 16	16,384	0.860	7.59	1.1	1.7
VGG-16 on CIFAR-8/10							
13	512	1 × 1	512	0.961	0.63	1	2.2
10	512	2 × 2	2048	0.958	2.17	2.8	1.4
7	256	4 × 4	4096	0.954	4.07	1.2	1.6
4	128	8 × 8	8192	0.890	12.4	1.4	1.8
2	64	16 × 16	16,384	0.783	13.0	1.3	1.8
VGG-16 on CIFAR-10/10							
13	512	1 × 1	512	0.94	1.5	1	2.8
10	512	2 × 2	2048	0.94	3.8	3.2	1.6
7	256	4 × 4	4096	0.93	6.4	1.3	1.6
4	128	8 × 8	8192	0.85	18.3	1.4	2.1
2	64	16 × 16	16,384	0.72	19.6	1.3	2.1

The results of VGG-16 trained on $K=3, 6, 8,$ and $10$ labels, namely CIFAR-K/10 (Supplementary Information).

### Applying filter cluster connections (AFCC)

The new comprehensive understanding of how the filters function in a trained deep architecture can promote improved technological implementation methods by applying filter’s cluster connections (AFCC) (Fig. 1). As each filter consists of only several small clusters, thereby generating a significant output signal for a small set of labels, its output for any other label can be neglected and the same accuracy can be achieved. To test the AFCC hypothesis a trained VGG-16 on CIFAR-100 was examined, where the accuracy of approximately $0.752$, is saturated at the 10th layer (Table 1). The number of weights of the FC layer is ${204,800}$; $512\times 2\times 2$ input units emerging from the $512$ filters multiplied by $100$ output units. All these weights which did not belong to a cluster in a specific filter were set to zero, resulting in approximately ${194,000}$ zeroed weights out of ${204,800}$(a $95\%$ reduction). The remaining ${10,800}$ weights is well approximated by $512\cdot 2\cdot 2\cdot {C}_{s}\cdot {N}_{c}\approx {10,600}$ (Table 1). After only a few training epochs, while maintaining the $\sim {194,000}$ zeroed weights as zero, the similar accuracy, $\sim 0.752,$ was recovered, which indicates that the FC layer can be significantly reduced and yield similar results (Supplementary Information). Note that the same filter clusters were detected for both training and test sets^18^. The performance of the same classification tasks with a significantly smaller amount of weights of the FC layer can improve the test computational complexity, as well as reduce the memory usage. Thus, the expansion of the AFCC method to include several layers can significantly reduce the complexity and deserves further research.

A similar effect was observed for EfficientNet-B0 trained on CIFAR-100, with an accuracy of $0.867$ (Table 2). The number of weights of the FC layer is ${128,000}$; $1280\times 1$ input units emerging from the $1280$ filters multiplied by $100$ output units. All of these weights that did not belong to a cluster in a specific filter were set to zero, resulting in $4900 (\sim 1280\cdot {C}_{s}\cdot {N}_{c}$) non-zero weights only (a $\sim 96\%$ reduction). After retraining the entire network with the same parameters, while including the $4900$ non-zeroed weights only, the accuracy increased to $\sim 0.873$, indicating that the FC layer can be significantly reduced and still yield similar or even increased accuracy (Supplementary Information). One cannot exclude a similar increase in accuracy without pruning the FC layer and using different training parameters, however, AFCC training is more efficient. This gain in the test computational complexity is expected to be enhanced further in datasets with a higher number of labels, such as ImageNet, and larger classification tasks.

The training of EfficientNet-B0 on CIFAR-100 indicates almost identical accuracies for stages $4$ and $5$ (Tables 2, 5) whereas the noise, $n,$ is non-monotonic between stages $3$ and $4$ for EfficientNet-B0 trained on ImageNet (Table 3). These results hint that stages $3{-}5$ of EfficientNet-B0 might be further optimized. Indeed, reducing the number of layers constituting stages $3$ and $4$ to one and training this modified EfficientNet-B0 on CIFAR-100 using transfer learning^26,27^, resulted in an accuracy $\sim 0.864$, which approached the original accuracy (Table 2). Similarly, reducing the number of layers in stage $5$ from $3$ to $2$, resulted in an accuracy of at least $0.862$ (Supplementary Information). Hence, following the proposed method, the latency of EfficientNet-B0 can be reduced without practically affecting its performance, at least for the CIFAR-100 dataset. Another simplification is the removal of stage $9$ from the construction of EfficientNet-B0 and connecting stage $8$ with only $320$ filters to the output layer, using the AFCC method. In this case, the obtained accuracy is at least $0.868$, which slightly exceeds the accuracy of the entire model terminating with $1280$ filters for the classification of CIFAR-100 (Supplementary Information).

## Discussion

The underlying mechanism of DL was quantitatively examined for two deep architectures, namely VGG-16 and EfficientNet-B0, trained on the CIFAR-10, CIFAR-100, and ImageNet datasets. These examinations enabled the verification of the suggested underlying mechanism of DL with different architectures consisting of $16$ to over $150$ layers as well as with the number of output labels ranging over three orders of magnitude $[{3,1},000]$.

The first step of the proposed method involves quantifying the accuracy of each CL of a trained deep architecture using the following procedure with relatively low computational complexity: The entire deep architecture is trained to minimize the loss. The weights of the first specified number of trained layers are held unchanged and their output units are FC to the output layer. These output units of an intermediate hidden layer represent the preprocessing of an input using a partial deep architecture, and the FC layer is trained to minimize the loss. The test set results indicate that the accuracy increases progressively with the number of layers towards the output (Tables 1, 2, 3, 4, 5, 6).

The trained FC layer weights are used to quantify the functionality of each filter that belongs to its input layer. The single-filter performance is calculated when all weights of the FC layer are silenced, except for the specific weights that emerge from the single filter. At this point, the test inputs are preprocessed by the first given number of trained layers, but influence the ${N}_{l}$ output units, representing the labels, only through the small aperture of one filter. This procedure generates an $({N}_{l}, {N}_{l})$ matrix, where element $(i, j)$ represents the average fields that are generated by label $i$ test inputs on output $j$. This matrix is normalized by its maximal element, following which a Boolean clipped matrix is formed following a given threshold. Its permuted version forms diagonal clusters (Fig. 3), the sizes of which increase only slightly when a deep architecture is trained on a dataset with an increasing number of labels (Tables 2, 3). The diagonal elements of the clusters represent the signal, whereas their off-diagonal elements represent the internal noise, resulting in uncertainty regarding the input label given an above-threshold output. The second type of noise, namely the external noise, stems from the above-threshold elements out of the diagonal clusters. This noise progressively decreases with the number of layers and forms the underlying mechanism of DL.

The proposed method suggests quantitative measures and building blocks to describe the underlying mechanism of DL. The vocabulary is the preferred subset of labels of each filter clusters, which compete with the filter’s noise. In addition to the contribution of this method to the understanding of how DL works, it provides insight into several practical aspects, including the following two. The first one is the possibility of improving the computational complexity and accuracy of deep architectures, and the second one is identifying weak stages in the construction of pre-existing deep architectures.

Using the single filter performance can lead to an efficient way to dilute the system without affecting its performance, as demonstrated by the AFCC method. Its expansion to include several layers can significantly reduce the complexity and deserves further research. This insightful dilution technique should be explored further on other datasets and deep architectures. In addition, its efficiency should be compared with that of other methods that primarily rely on random dilution processes^28–31^ and assess their effectiveness in reducing complexity.

The presented universal underlying mechanism of DL may suggest an estimation method for the necessary number of filters in each layer. Each label must appear at least once in the clusters of the layer, hence, $1280$ filters in stage $9$ of EfficientNet-B0 appear to be insufficient to classify, for example, ${100{,}000}$ labels. Nevertheless, the results indicate that the number of diagonal elements, ${C}_{s}\cdot {N}_{c}$, increases from $3.6$ for CIFAR-100 to $15.4$ for ImageNet (Tables 2, 3). Therefore, one cannot exclude the reality in which the filters constitute many relatively small clusters when the number of labels increases further. In addition, the information that is embedded in a single filter, namely clusters and noise, suggests procedures for pruning or retraining inefficient filters, such as highly noisy or low output-field filters. These procedures may improve the accuracy with reduced computational complexity and latency in the test phase, however, the investigation thereof requires further research.

### Supplementary Information

Supplementary Information.

## Data Availability

Source data are provided in this study, including all data supporting the plots, along with other findings of this study.
