# Supplementary material for: Towards a universal mechanism for successful deep learning
Source: Sci Rep. 2024 Mar 11;14:5881. doi: 10.1038/s41598-024-56609-x (PMC10928127; doi:10.1038/s41598-024-56609-x)
Supplement: Supplementary file 1 — Supplementary Information. [file 41598_2024_56609_MOESM1_ESM.pdf]

Supplementary Information

Towards a universal mechanism for successful deep learning

Yuval Meir[1,+], Yarden Tzach[1,+], Shiri Hodassman[1], Ofek Tevet[1] and Ido Kanter[1,2*]

[1]Department of Physics, Bar-Ilan University, Ramat-Gan, 52900, Israel.

[2]Gonda Interdisciplinary Brain Research Center, Bar-Ilan University, Ramat-Gan, 52900, Israel.

[+] These authors contributed equally

[*]Corresponding author email: ido.kanter@biu.ac.il

Architectures and Training the fully connected layer. Two different architectures were examined. VGG-16[1]and EfficientNet-B0[2]. Both architectures were trained to classify the CIFAR-10 and CIFAR-100 datasets, as well as subclasses of their labels. In addition, EfficientNet-B0 was trained to classify the ImageNet dataset. Both architectures were trained with no biases on the output units. This was done to assure that each filter's effect on the output fields will be exemplified and will not be overshadowed by the much larger biases. Removing the biases of the output layer did not affect the architectures' average accuracies, in comparison to architectures trained with output biases.

The examination process was done by taking each architecture at designated layers and training a fully connected (FC) layer between the output of that specific layer and the output layer, corresponding to the labels. During training, only the FC layer was trained, while weights and biases of the rest of the architecture remain fixed. For VGG-16 the input units to the FC layers were selected after the max-pooling operations adjacent to layer 2, 4, 7, 10, and 13. For EfficientNet-B0, stages 1, 3, 4, 5, 7 and 9 which reduce the input size due to the stride-2 were examined.

For each examined layer, m, the output of the training set for the mth layer was used as a preprocessed dataset to train the FC layer. For each architecture, optimized hyper-parameters were used for the examined layers.

Data preprocessing. For VGG-16, each input pixel of an image (32×32) from the CIFAR-10 and CIFAR-100 databases was divided by the maximal pixel value, 255, multiplied by 2, and subtracted by 1, such that its range was $[-1, 1]$. In all simulations, data augmentation was used, derived from the original images, by random horizontally flipping and translating up to four pixels in each direction.

For EfficientNet-B0, the images were normalized by subtracting the average value of each color and dividing by its standard deviation. This varies by the size of the training set that was used, which change based on the number of different labels trained, K. For CIFAR-K/10 and CIFAR-K/100 the images were also expanded from their initial size of (32×32) to (224×224)[3]. For all datasets, data augmentation was also used, which included a random horizontal flip, a random rotation of up to two degrees, a random translation of the image of up to four pixels in each direction and a shear of up to two degrees.

Optimization. The cross-entropy cost function was selected for the classification task and was minimized using the stochastic gradient descent algorithm[4,5]. The maximal accuracy was determined by searching through the hyper-parameters (see below). Cross-validation was confirmed using several validation databases, each consisting a fifth of the training set examples, randomly selected. The averaged results were in the same standard deviation (Std) as the reported average success rates. The Nesterov momentum[3] and L2 regularization method[4] were applied.

Hyper-parameters. The hyper-parameters η (learning rate), μ (momentum constant[6]), and α (regularization L2[4]) were optimized for offline learning, using a mini-batch size of 100 inputs. The learning rate decay schedule[5,7] was also optimized. A linear scheduler was used such that it was multiplied by the decay factor, q, every $\Delta\mathrm{t}$ epochs, and is denoted below as $(\mathrm{q}, \Delta\mathrm{t})$. Different hyper-parameters were used for each one of the architectures on each classification task.

Datasets. The used datasets were CIFAR-10, CIFAR-100 and ImageNet. Tests on the systems were extended by creating smaller datasets of K labels, chosen form the CIFAR-10 and CIFAR-100 datasets. For CIFAR-100, $K = 20, 40, 60,$ and 100 were used where for each progressively increasing K the previous subset is included, e.g. the labels chosen for $K = 20$ are included in $K = 40$, and for CIFAR-10, $K = 3, 6, 8,$ and 10 were used. The CIFAR-K/10 and CIFAR-K/100 were normalized like CIFAR-10 and CIFAR-100 respectively for each architecture.

For ImageNet, 10,000 images, 10 images per label, were selected out of the validation set as the test dataset and the remaining 40,000 as training images.

VGG-16 Hyper-parameters.

VGG-16 was trained using the following hyper-parameters to reach maximal accuracies on CIFAR-K/100:

**VGG-16**			
**CIFAR-20/100**			
η	μ	α	epochs
0.004	0.965	3e-3	300

CIFAR-40/100			
η	μ	α	epochs
0.002	0.975	4e-3	300
**CIFAR-60/100**			
η	μ	α	epochs
0.002	0.975	4e-3	300
**CIFAR-100**			
η	μ	α	epochs
0.002	0.975	4e-3	300

Where the decay schedule for the learning rate is:

$$(\mathrm{q}, \Delta\mathrm{t}) = (0.65, 20)$$

For the training of the FC layer. Each layer m was FC to the K outputs via a FC layer of size $N(m) \cdot K$. The FC layer was trained using the hyper-parameters: $\eta = 0.005$, $\mu = 0.975$, $\alpha = 1.5e - 5$, with a learning rate scheduler of $q = 0.65$ every 20 epochs while the rest of the system's weight values and biases remained fixed.

VGG-16 was trained using the following hyper-parameters to reach maximal accuracies on CIFAR-K/10:

VGG-16			
**CIFAR-K/10**			
η	μ	α	epochs
0.01	0.975	0.0015	200

Where the decay schedule for the learning rate is:

$$(\mathrm{q}, \Delta\mathrm{t}) = (0.65, 20)$$

For the training of the FC layer, $\eta = 0.02$, $\mu = 0.995$, $\alpha = 1e - 7$, with a learning rate scheduler of $q = 0.6$ every 20 epochs while the rest of the architecture's weight values and biases remained fixed.

EfficientNet-B0 Hyper-parameters.

EfficientNet-B0 was trained on CIFAR-K/100 and ImageNet datasets using transfer learning[8] on the pre-trained EfficientNet-B0 on ImageNet dataset. The transfer learning was done using the following hyper-parameters and learning rate scheduler:

**EfficientNet-B0**			
**CIFAR-K/100**			
η	μ	α	epochs
0.01	0.9	0.001	200

Where the decay schedule for the learning rate is:

$$(\mathrm{q}, \Delta\mathrm{t}) = (0.975, 1)$$

For the first seven stages, the learning rate η was multiplied by a factor of 0.1, and for the last stage by 0.2.

The output of each layer m was sampled by a 7×7 average-pooling and then FC to the K outputs via a FC layer of size $N(m) \cdot K$. The FC layer was trained using the hyper-parameters: $\eta = 0.005$, $\mu = 0.975$, $\alpha = 1.5e - 5$, with a learning rate scheduler of $q = 0.975$ every epoch while the rest of the architecture's weight values and biases remained fixed.

The training of EfficinetNet-B0 with reduced number of layers in stages 3 and 4 to 1, was done by using the hyper-parameters: $\eta = 0.002$, $\mu = 0.98$, $\alpha = 1e - 4$.

The training of EfficinetNet-B0 with reduced number of layers in stage 5, from 3 to 2, was done by using the hyper-parameters: $\eta = 0.01$, $\mu = 0.965$, $\alpha = 5e - 4$.

Explanations for Figure 1. In Fig 1 Left column, For VGG-16 on CIFAR-100, the 100 output fields of each filter were summed over all $10{,}000$ inputs of the test set, resulting in a $100x100$ matrix where each cell (i, j) represents the summed field of output field j for all test set inputs of label i. The matrix was then normalized by dividing by its maximal value, resulting in each matrix having a maximal value of 1. In the center column the clipped Boolean output field matrix is displayed, where each element whose value is above a threshold (0.3) is set to 1 and all others are zeroed.

In the right column, the axes are permuted such as all labels belonging to a cluster are grouped together consecutively, thereby displaying the clusters in an adjacent fashion where they are displayed as a diagonal block of elements with value 1. Each cluster is defined as a subset of n indices where for each $i, j \in n$ elements (i, j) have the value of 1. The size of the cluster is defined as n^2 where n is the number of labels whose all possible pair permutations form the cluster, where the minimal size can be 1, that is one element on the diagonal or 100, the entire matrix. The elements that are equal to 1 are then colored as white, representing that they belong to a cluster in the filter, while non-cluster cells with the value of 1 are classified as above-threshold external noise and are colored yellow.

The calculation of the clusters was done by running along the diagonal, from index $(0,0)$ to $(99,99)$ where the first (i, i) element to have a value of 1 is initially designated as a cluster of size $1x1$. The next (j, j) $where\ j \neq i$ element to have a value of 1 is then checked to see if can complete a cluster with (i, i), if yes, then it is added to the cluster and the next diagonal element to have a value of 1 is checked if completes a cluster with i and j, if yes it is appended to the cluster, if not the system continues to the next cell. This process is repeated for all value 1 cells in the diagonal as long as there are elements who do not belong to a cluster. Note that this process is not uniquely defined, the order by which the indices are iterated can change the outcome of the clustering process, such as a filter with two clusters of sizes $3x3$ and $1x1$ retrieved by iterating from 0 to 99 can yield in certain very rare scenarios, two clusters of size $2x2$. While possibly alternating the results of a single filter, the overall obtained averaged results remain the same when performing the cluster creation while iterating in a reversed order, since those scenarios are very rare and occur in a negligible number of filters.

The external noise is calculated for each filter as the elements with value 1 who do not belong to any cluster. They can be seen in color yellow in the right column.

Explanations for Figure 2. A. The clipped binary signal per label was obtained by the diagonal signal of the summation of all binary clipped matrices of the filters together. The $noise_I$, the average internal noise of each label, is equal to the sum of all non-diagonal elements belonging to a cluster, on that label's row. The external noise, $noise_E$, of each label is equal to the sum of all non-diagonal elements not belonging to a cluster, on that label's row. **B.** Similar to **A** but now the signal, internal

noise and external noise were calculated by the original accumulated fields of the filters and not the clipped binary fields. The internal and external noise were summed by their obtained unit indices from the binary clipped matrix.

Figure 3. Test error for VGG-16 trained on CIFAR-K/100 The error rate of VGG-16 on CIFAR-K/100 was tested with $K = 20, 40, 60$ and 100, where the subset for the lowest value of K labels were randomly chosen and then for progressively increasing K the previous K labels were included, e.g. the labels chosen for $K = 20$ are included in $K = 40$. The K labels were chosen uniformly from the 20 super-classes of the dataset.

Figure 4. Test error for EfficientNet-B0 trained on CIFAR-K/100 The error rate of EfficientNet-B0 on CIFAR-K/100 was tested with $K = 20, 40, 60$ and 100, as done in Figure 3. The slope of the fitted line for the accuracies was 0.0013. This process was repeated for 5 different subsets and the slopes fluctuated in the range [0.0012, 0.0013].

Figure 5. Test error for VGG-16 trained on CIFAR-K/10 The error rate of VGG-16 on CIFAR-K/10 was tested with $K = 3, 6, 8$ and 10, where the subset for the lowest value of K labels were randomly chosen and then for progressively increasing K the previous K labels were included, e.g. the labels chosen for $K = 3$ are included in $K = 6$.

Applied Filter's Cluster Connections (AFCC). Optimization of the system can be achieved by capitalizing on the nature of the filter clusters. First, each output unit only receives a signal from filters whose clusters contain that output label, meaning that diluting all other unnecessary connections can reduce the complexity of the system. Second, filters can be trained by inputs whom belong to their clusters, thereby lowering the complexity of the training as well as the number of computational tasks needed. The first point is exemplified in the Discussion, where a trained FC was connected to the 10th layer, yielding an accuracy of ~ 0.752 and all weights who connect an output unit with a filter that does not constitute its filter are set to zero. After zeroing all FC weights, a short training section of a few epoch ~ 10 is commenced, where the zeroed weights remain zero and only the FC and 10th convolution layer are trained. The hyper-parameters used were $\eta = 0.00001$, $\mu = 0.9$, $\alpha = 1.5e - 2$. A similar effect was observed for EfficientNet-B0 on CIFAR-100 where each output unit only receives a

signal from filters whose clusters contain that output label, but also the entire network was further trained, yielding an accuracy of ~0.873. The hyper-parameters used were $\eta = 0.005$, $\mu = 0.975$, $\alpha = 1e - 4$. For the first seven stages, the learning rate η was multiplied by a factor of 0.1, and for the last stage by a factor of 0.2. The decay schedule for the learning rate was $(\mathrm{q}, \Delta\mathrm{t}) = (0.975, 1)$.

Statistics. Statistics for all results of EfficientNet-B0 were obtained using at least five samples. For VGG-16, results on CIFAR-K/10 were obtained using five samples, while for CIFAR-K/100 results were obtained using at least four samples.

Hardware and software. We used Google Colab Pro and its available GPUs. We used Pytorch for all the programming processes.

1. Simonyan, K. & Zisserman, A. Very Deep Convolutional Networks for Large-Scale Image Recognition. (2014).
2. Tan, M. & Le, Q. V. EfficientNet: Rethinking Model Scaling for Convolutional Neural Networks. (2019).
3. Keys, R. Cubic convolution interpolation for digital image processing. *IEEE Trans. Acoust.* **29**, 1153–1160 (1981).
4. Schmidhuber, J. Deep learning in neural networks: An overview. *Neural Networks* **61**, 85–117 (2015).
5. He, K., Zhang, X., Ren, S. & Sun, J. Deep Residual Learning for Image Recognition. (2015).
6. Botev, A., Lever, G. & Barber, D. Nesterov's accelerated gradient and momentum as approximations to regularised update descent. in *2017 International Joint Conference on Neural Networks (IJCNN)* 1899–1903 (IEEE, 2017). doi:10.1109/IJCNN.2017.7966082
7. You, K., Long, M., Wang, J. & Jordan, M. I. How Does Learning Rate Decay Help Modern Neural Networks? (2019).
8. Yosinski, J., Clune, J., Bengio, Y. & Lipson, H. How transferable are features in deep neural networks? (2014).
